# Effect of Sacubitril-Valsartan on Restoration and Maintenance of Sinus Rhythm in Patients With Persistent Atrial Fibrillation

**DOI:** 10.3389/fcvm.2022.870203

**Published:** 2022-05-30

**Authors:** Qingsong Chen, Yunlin Chen, Fang Qin, Huaan Du, Chunxia Gan, Bei Zhou, Na Wang, Mingyang Xiao, Zhenhong Ou, Wei Zhao, Ben Cui, Zengzhang Liu, Yuehui Yin

**Affiliations:** Department of Cardiology, The Second Affiliated Hospital of Chongqing Medical University, Chongqing, China

**Keywords:** atrial fibrillation, rhythm, electrical cardioversion, sacubitril-valsartan, treatment

## Abstract

**Background:**

Existing studies have shown that sacubitril-valsartan ameliorated atrial remodeling in atrial fibrillation (AF) and favored maintenance of sinus rhythm in patients with AF and heart failure. However, the effect of sacubitril-valsartan in patients with persistent AF is yet unknown. We aimed to evaluate the effect of sacubitril-valsartan on restoration and maintenance of sinus rhythm in patients with persistent AF who underwent electrical cardioversion (ECV).

**Method:**

Consecutive patients with persistent AF who underwent ECV between 1 January 2016 and 30 September 2020 were investigated in this retrospective cohort study. All eligible patients were categorized into sacubitril-valsartan users and sacubitril-valsartan non-users based on whether they received treatment with sacubitril-valsartan or not. The endpoint was ineffictive ECV, defined as the composite of failure to terminate AF or any recurrence of AF during 30 days follow-up.

**Results:**

A total of 76 patients were enrolled in this study, including 28 sacubitril-valsartan users and 48 non-users. Within a follow-up of 30 days after ECV, the endpoint had occurred in 7 (25%) of 28 sacubitril-valsartan users and 25 (52%) of 48 non-users. Significantly lower rate of ineffictive ECV in sacubitril-valsartan users compared with non-users was shown in Kaplan-Meier survival curves (*P* = 0.02; Log-rank test). Multivariate Cox regression analysis indicated that sacubitril-valsartan use (hazard ratio [HR], 0.35; 95% confidence interval [CI], 0.14–0.91), amiodarone use (HR, 0.32; 95% CI, 0.13–0.78), left atrial diameter ≤ 39 mm (HR, 0.21; 95% CI, 0.06–0.71) were independently associated with a decreased rate of ineffective electrical cardioversion.

**Conclusion:**

Use of sacubitril-valsartan is associated with a significantly decreased risk of ineffective ECV compared with non-users in patients with persistent AF.

## Introduction

Atrial fibrillation (AF), the most frequent type of clinical tachyarrhythmia, is correlated with an increased risk of stroke and heart failure (HF). Rate-control and rhythm-control therapy are two major therapeutic strategies for AF. Recently, studies have demonstrated that rhythm-control therapy can improve AF-related symptoms, prevent AF progression (1) and even reduce the risk of cardiovascular complications (2). The rhythm-control strategy may refer to a combination of treatment approaches, including antiarrhythmic medication, catheter ablation and electrical cardioversion (ECV) (3). Of these, ECV is recommended as first-line medical therapy for symptomatic patients with persistent AF (3). However, some studies reported ECV failed to restore sinus rhythm in 12–26% of persistent AF patients (4, 5), and the rate of AF recurrence is up to 57–63% within 30 days after ECV (6). Thus, there is a unmet need to identify better regimes on restoration and maintenance of sinus rhythm in patients with persistent AF who underwent ECV.

The electrical and structural atrial remodeling, characterized by atrial action potential duration (APD) shortening, reduction in atrial effective refractory period (ERP), atrial enlargement and fibrosis, is a crucial mechanism in maintenance and progression of AF (7–9). Increased left atrial size has been confirmed to be strongly associated with the failure of ECV in many studies (4, 10, 11). Renin-angiotensin-aldosterone system (RAAS) was involved in electrical and structural atrial remodeling during AF and development and progression of AF may be prevented by inhibiting the activity of the RAAS system (12, 13). Natriuretic peptides (NPs), including atrial natriuretic peptide (ANP) and brain natriuretic peptide (BNP), were found to play a role in AF (14). Previous study reported that recombinant human ANP could reduce the occurrence of postoperative AF (15).

Sacubitril-valsartan, a first-in-class angiotensin receptor neprilysin inhibitor (ARNI), has been shown to act on both RAAS and NPs (16). In 2012, the PARAMOUNT trial initially demonstrated a reduction in left atrial size after administration of sacubitril-valsartan in patients with heart failure with preserved ejection fraction (HFpEF) (17). Furtherly, experimental and clinical studies uncovered that sacubitril-valsartan could ameliorate atrial remodeling in AF (18) and favor maintenance of sinus rhythm in patients with AF and HF (19–21). Nevertheless, the effect of sacubitril-valsartan in patients with persistent AF is yet unknown. The aim of this study is to evaluate the effect of sacubitril-valsartan on restoration and maintenance of sinus rhythm in patients with persistent AF who underwent ECV.

## Materials and Methods

### Study Design and Participants

In this single-center, retrospective cohort study, we retrieved patient-level demographic and clinical data from the electronic medical record (EMR) system between 1 January 2016 and 30 September 2020. Deidentified information recorded in the EMR included demographic characteristics, comorbidities, symptoms, laboratory and imaging findings, drug prescriptions, ECV procedures, etc. Our study adhered to the Declaration of Helsinki guidelines. According to International Ethical Guidelines for Health-Related Research Involving Humans (22), The Research Ethics Committee of the Second Affiliated Hospital of Chongqing Medical University (Chongqing, China) approved the study and granted a waiver of informed consent. Patients aged at least 18 years were eligible for inclusion if they had a diagnosed persistent AF and received ECV. Persistent AF was defined as AF lasting more than 7 days or needing cardioversion for termination. We excluded individuals if they had: (1) Any of the following comorbidities including acute coronary syndrome, acute heart failure, rheumatic valvular heart disease, hypertrophic cardiomyopathy, dilated cardiomyopathy, or hyperthyroidism. (2) Previous cardiac surgery or AF catheter ablation within 3 months. (3) Showing symptomatic sinus bradycardia or junctional escape rhythm after ECV. (4) Any of new prescription or discontinuation of sacubitril-valsartan, amiodarone, or angiotensin-converting enzyme inhibitors (ACEI)/angiotensin receptor blockers (ARB) monotherapy during the follow up period.

### Group Assignment

Eligible patients were categorized as sacubitril-valsartan users and sacubitril-valsartan non-users.

As documented in the EMR, sacubitril-valsartan was tried to modify the atrial substrate in patients with persistent AF. Sacubitril-valsartan users had received initiation (initial dosage, 50 or 100 mg twice daily) and uptitration (a target dosage of 200 mg twice daily or tolerated dose) regimens for sacubitril-valsartan, for at least 2 weeks before ECV.

### Electrical Cardioversion Procedure

All patients underwent transesophageal echocardiography 24 h prior to ECV procedure to rule out the possibility of thrombus in the left atrium and left atrial appendage. Blood pressure, cardiac rhythm, and oxygen saturation were continuously monitored during the procedure. After sedation with diazepam, pads were positioned in antero-lateral configuration and transthoracic ECV was performed with biphasic shock using 200 J initially. If need, energy increase to 300 J. If AF persisted after two shocks, no more attempts of ECV were repeated.

### Follow Up

The follow-up time was defined as the time from the beginning of ECV (day 1) to the date of 30 days after ECV. Anticoagulation was continued for at least 4 weeks after ECV. After the ECV procedure, all patients were hospitalized with continuous cardiac rhythm monitoring till discharge. A 12-lead electrocardiogram (ECG) or 24-h Holter recording was performed at 30-day follow-up and at any time the patient experienced irregular pulses or complained of palpitations or any other symptoms possibly related to the recurrence of AF after discharge.

### Outcome

The endpoint of this study was ineffective ECV, defined as the composite of failure to terminate AF or any recurrence of AF during 30 days follow-up. Failure to terminate AF was defined as failure to achieve the presence of at least two consecutive sinus complexes after ECV. Recurrence of AF was defined as any episode of atrial tachycardia, atrial flutter or AF lasting at least 30 s confirmed by a 12-lead ECG or 24-h Holter after successful termination of AF.

### Statistical Analysis

Continuous variables are presented as mean ± standard deviation (SD) and compared using independent-samples Student’s *t*-test if normally distributed. For continuous variables with a non-normal distribution, data are presented as median (interquartile range) and compared using the Mann-Whitney *U* test. The normality of data was assessed by Shapiro-Wilk normality test. Categorical variables are presented as percentage and compared with Chi-square or Fisher’s exact test. Survival curves were constructed using the Kaplan-Meier method and compared by the log-rank test. Univariate and multivariate analysis were performed using the Cox proportional hazards model. Variable selection for multivariate Cox regression model was based on sample size, variables considered to be clinically relevant and those with a *p*-value of <0.1 in univariate analysis. The receiver operating characteristic (ROC) analysis was performed to dichotomize continuous variables. If the area under the ROC curve (AUC) was greater than 0.5, the optimal cutoff value was defined by selecting the maximum Youden index. Otherwise, the optimal cutoff value was defined according to threshold of clinical significance. A *p*-value of <0.05 was considered statistically significant. All analysis were performed using R software (R version 4.0).

## Results

**FIGURE 1:**
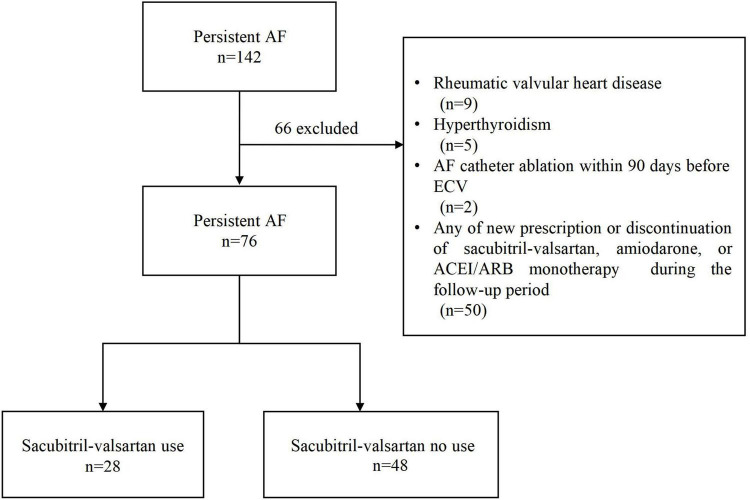
Flowchart of the study. AF, atrial fibrillation; ECV, electrical cardioversion; ACEI, angiotensin-converting enzyme inhibitors; ARB, angiotensin receptor blockers.

Among the 142 patients, a total of 76 patients were enrolled in this study based on the inclusion and exclusion criteria, including 28 sacubitril-valsartan users and 48 non-users (Figure 1). The median age of all eligible patients was 67.0 (60.0–72.0) years, and 48 (63%) were male. None of the patients received cardiac glycosides or non-dihydropyridine calcium channel blockers therapy. Table 1 summarizes the differences in demographics and clinical characteristics among the two groups. Overall, sacubitril-valsartan users were not treated with either ACEI or ARB monotherapy and seemed to have a larger left atrial diameter (LAD) (*P* = 0.04), a lower serum concentration of serum Na+ (*P* < 0.001) and N-terminal pro-B-type natriuretic peptide (NT-proBNP) (*P* = 0.04), be less frequently treated with beta-blocker (*P* = 0.03). The remaining baseline characteristics between two groups were generally similar.

**TABLE 1:** Characteristics of the patients with persistent atrial fibrillation at baseline.^a^

	All patients	Sacubitril-valsartan use		*P*-value
	(*n* = 76)	No (*n* = 48)	Yes (*n* = 28)	
Sex				0.74
Female	28 (37)	17 (35)	11 (39)	
Male	48 (63)	31 (65)	17 (61)	
Age, years	67.0 (60.0–72.0)	66.5 (58.8–73.0)	67.0 (62.0–72.0)	0.87
BMI, kg/m^2^	24.2 (21.7–26.7)	24.7 (22.0–27.7)	23.5 (21.4–25.1)	0.24
Smoking	30 (40)	18 (38)	12 (43)	0.65
Alcohol	30 (40)	18 (38)	12 (43)	0.65
Hypertension	42 (55)	28 (58)	14 (50)	0.48
CAD	18 (24)	14 (29)	4 (14)	0.14
Diabetes	6 (8)	5 (10)	1 (4)	0.40
AF duration, months	12.0 (2.0–48.0)	18.0 (2.0–51.0)	12.0 (2.0–48.0)	0.45
EHRA score				0.08
II	19 (25)	8 (17)	11 (39)	
III	37 (49)	27 (56)	10 (36)	
IV	20 (26)	13 (27)	7 (25)	
NYHA functional class				0.93
I	10 (13)	7 (15)	3 (11)	
II	56 (74)	35 (73)	21 (75)	
III	10 (13)	6 (12)	4 (14)	
LAD, mm	43.0 (40.0–47.0)	42.5 (39.0–44.3)	44.0 (41.8–48.5)	0.04
LVEF, %	64.0 (56.0–70.0)	64.0 (58.0–70.5)	64.5 (51.8–69.3)	0.57
eGFR, mL/min. 1.73 m^2^	81.0 (67.9–93.7)	84.5 (74.4–92.2)	75.1 (62.0–94.0)	0.08
Serum Na+, mmol/L	140 (138–142)	141 (139–143)	138 (137–140)	<0.001
Serum K+, mmol/L	3.97 (3.80–4.22)	3.95 (3.72–4.18)	4.01 (3.83–4.35)	0.18
NT–proBNP, pg/mL	1,030 (604–1,660)	1,370 (641–1,770)	692 (478–1,270)	0.04
Amiodarone use	64 (84)	37 (77)	27 (96)	0.06
ACEI/ARB monotherapy	25 (33)	25 (52)	0 (0)	<0.001
ACEI use	15 (20)	15 (31)	0 (0)	0.01
ARB monotherapy	10 (13)	10 (21)	0 (0)	<0.001
Beta-blocker use	22 (29)	18 (38)	4 (14)	0.03
^b^Length of monitoring, days	4.00 (3.00–5.25)	3.50 (3.00–5.25)	4.00 (2.75–5.25)	0.90

*BMI, body mass index; CAD, coronary artery disease; AF, atrial fibrillation; EHRA, European Heart Rhythm Association; NYHA, New York Heart Association; LAD, left atrial diameter; LVEF, left ventricular ejection fraction; eGFR, estimate glomerular filtration rate; NT-proBNP, N-terminal pro-B-type natriuretic peptide; ACEI, angiotensin-converting enzyme inhibitors; ARB, angiotensin receptor blockers.*

*^a^Data are presented as number (percentage) or median (interquartile range) as appropriate.*

*^b^Length of monitoring is presented as the length of hospital stay after electrical cardioversion with monitoring.*

**FIGURE 2:**
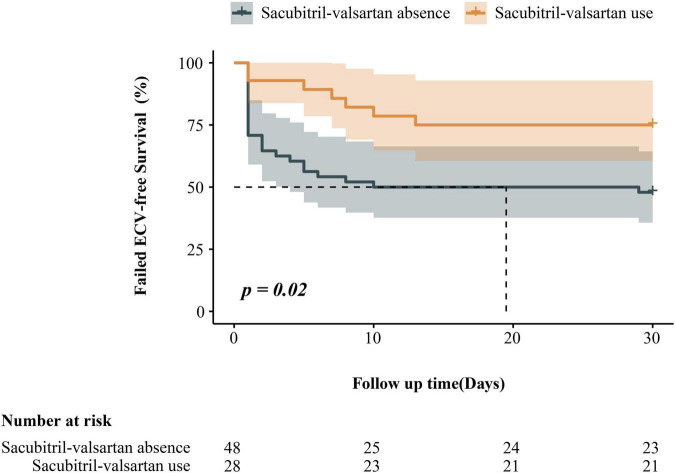
Kaplan–Meier estimates of the ineffective electrical cardioversion among patients with persistent atrial fibrillation, according to sacubitril-valsartan use.

After a follow-up of 30 days, the endpoint had occurred in 7 (25%) of 28 sacubitril-valsartan users and 25 (52%) of 48 non-users. A significantly lower rate of ineffictive ECV in sacubitril-valsartan users compared with non-users was shown in Kaplan-Meier survival curves (log-rank *P* = 0.02, Figure 2).

**FIGURE 3:**
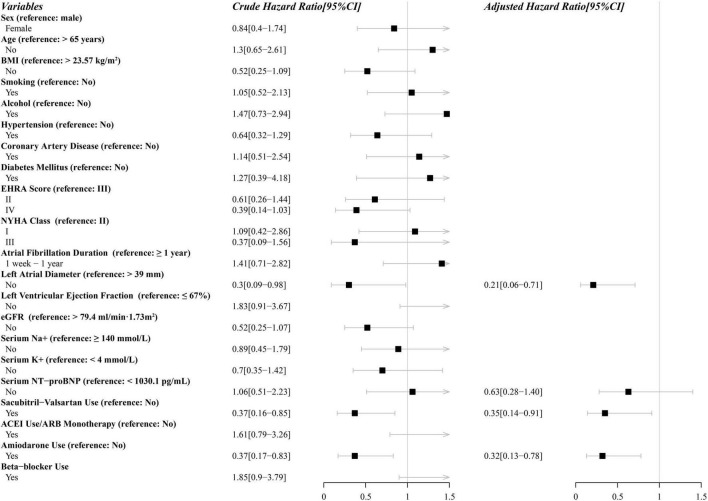
Parameters in uni- and multivariate analysis associated with ineffective electrical cardioversion performed with the Cox proportional-hazards model. CI, confidence interval; BMI, body mass index; EHRA, European Heart Rhythm Association; NYHA, New York Heart Association; eGFR, estimate glomerular filtration rate; NT-proBNP, N-terminal pro-B-type natriuretic peptide; ACEI, angiotensin-converting enzyme inhibitors; ARB, angiotensin receptor blockers.

Univariate Cox regression analysis of potential factors for ineffictive ECV is shown in Figure 3. Sacubitril-valsartan, amiodarone, LAD, NT-proBNP were finally included in the multivariate Cox proportional hazard regression analysis. After adjusting for covariates, multivariate Cox regression analysis indicated that sacubitril-valsartan use (hazard ratio [HR], 0.35; 95% confidence interval [CI], 0.14–0.91), amiodarone use (HR, 0.32; 95% CI, 0.13–0.78), left atrial diameter ≤ 39 mm (HR, 0.21; 95% CI, 0.06–0.71) were independently associated with a decreased rate of ineffective electrical cardioversion. Complete multivariate model is presented in Figure 3.

## Discussion

The main findings of our retrospective cohort study showed that use of sacubitril-valsartan was associated with a significantly decreased risk of ineffictive ECV in patients with persistent AF who underwent ECV. Additionally, we also found amiodarone use, LAD ≤ 39 mm were independent factors for ECV success.

Previous studies on sacubitril-valsartan mainly focused on the fields of HF and hypertension. Recently, several studies with preliminary clinical evidence have manifested potential antiarrhythmic effects of sacubitril-valsartan on AF (19–21, 23, 24). Two studies reported that sacubitril-valsartan was favorable for restoration and maintenance of sinus rhythm in cases with AF and HF (19, 20). In a recent study, De Vecchis et al. (21) showed that patients with chronic heart failure receiving sacubitril-valsartan therapy had significantly less episodes of AF recurrence and a significantly higher increase in average peak atrial longitudinal strain (26.5 vs. 22.5%), when compared to those who taking ACEI/ARB monotherapy. Russo et al. (23) demonstrated that, in dilated cardiomyopathy (DCM) patients with reduced ejection fraction who had a dual-chamber implantable cardioverter defibrillator (ICD), sacubitril-valsartan treatment was associated with a significant reduction in AF episodes (34 vs. 19, *P* = 0.03) and improvement in P wave sensing, atrial pacing threshold during a 12-month follow-up. Similarly, De Diego and colleagues observed that there was a trend for a reduction of paroxysmal atrial tachycardia or AF episodes (from 14 to 10%) in patients with HFrEF and ICD after the sacubitril-valsartan regimen (24). All of the above studies focused on patients with both AF and HF. To the best of our knowledge, this is the first study to investigate the role of sacubitril-valsartan in patients with persistent AF who underwent ECV. However, the exact mechanisms underlying such beneficial effects of sacubitril-valsartan on reduction in the risk of ineffictive ECV are not completely understood.

A limited number of studies suggest that inhibiting atrial electrical and structural remodeling, and facilitating the cardiac electro-mechanical reverse remodeling may be potential mechanisms of sacubitril-valsartan. In animal studies, Suo et al. (25) observed that as compared to ARB monotherapy, sacubitril-valsartan significantly attenuated left atrial fibrosis in a mice model. In a rabbit model (18), sacubitril-valsartan ameliorated the electrical remodeling of AF by inhibiting the reduction of L-type calcium current density and calcium overload, subsequently preventing atrial AERP and APD shortening. In addition, sacubitril-valsartan alleviated the structural remodeling of AF by inhibiting the up-regulation of collagen I and III levels, subsequently preventing atria fibrosis and enlargement (18). In the clinical setting, the PARAMOUNT trial demonstrated a greater reduction in left atrial size, indicative of reverse left atrial remodeling, in patients with HFpEF receiving sacubitril-valsartan compared with those receiving valsartan (17). Furthermore, a significant reduction in P wave dispersion and left atrial size was identified after treatment with sacubitril-valsartan (23, 26–28). Previous studies has shown that prolonged P wave dispersion was correlated with an increased risk of AF recurrence in persistent AF patient who underwent ECV (29, 30). Thus, we speculate that the effect of sacubitril-valsartan on the reduction in risk of ineffictive ECV might be associated with the reduction in P wave dispersion, which still requires to be confirmed by additional research.

Previous two randomized, open label studies have indicated that treatment with ACEI enalapril or ARB irbesartan in combination with amiodarone may reduce the risk of AF recurrence and facilitate long-term maintenance of sinus rhythm after ECV as compared with amiodarone alone in patients with persistent AF (31, 32). Meanwhile, the above studies (31, 32) mentioned that ACEI/ARB could prevent or modify atrial remodeling. Sacubitril-valsartan, contains the angiotensin receptor blocker valsartan and the neprilysin inhibitor prodrug sacubitril (16). This drug targets to inhibit the RAAS and decrease degradation of the NPs (33). NPs themselves exert many biological effects in the cardiovascular system, including anti-fibrosis, anti-inflammatory, inhibition of the renin-angiotensin and sympathetic systems (34, 35). Therefore, based on the pharmacological mechanisms of sacubitril-valsartan, it can be presumed that sacubitril-valsartan could also prevent or modify atrial remodeling through similar or even stronger effects as ACEI/ARB monotherapy. More recently, Carluccio et al. assessed changes in echocardiographically derived hemodynamic profiles induced by sacubitril-valsartan in a large consecutive series of patients with HF (36, 37). They found that an important role of sacubitril-valsartan on improvement cardiovascular hemodynamics including cardiac output and left ventricular filling pressure (36, 37), which might provide new insights into the underlying mechanism of effects of sacubitril-valsartan on restoration and maintenance of sinus rhythm for persistent AF.

Left atrial enlargement was proved to be an independent risk factor for AF recurrence in previous studies (4, 10, 11). The results of this study suggest that LAD ≤ 39 mm is associated with a significantly decreased risk of ineffictive ECV compared with that over 39 mm. Nevertheless, current evidence is inconclusive to define the optimal cutoff value of left atrial size in predicting AF recurrence (11, 38). Left atrial enlargement was associated with left atrial fibrosis, which is likely to result in regional conduction slowing and increased electrophysiological heterogeneity, subsequently providing a substrate for AF (39).

Results from clinical trials demonstrated that pretreated with antiarrhythmic drugs (AADs) before ECV could improve restoration and maintenance of sinus rhythm in persistent AF (40, 41), in agreement with our findings. In a recent meta-analysis of 8 studies, treatment with amiodarone was found to be associated with higher rates of restoration (relative risk [RR], 1.22; 95% CI, 1.07–1.39) and long-term maintenance (RR, 4.39; 95% CI, 2.99–6.45) of sinus rhythm (6).

A few limitations should be acknowledged in our study. First, the generalizability of our findings is only restricted to patients with persistent AF, and future studies are required to ascertain whether such benefits could be observed in paroxysmal AF. Second, continuous monitoring of cardiac rhythm after discharge was not feasible, and that might has resulted in missed asymptomatic episodes of AF, which might be a source of information bias. Third, the follow-up duration of this study was relatively short, and effect of sacubitril-valsartan on long-term maintenance of sinus rhythm awaits further exploration. Fourth, our data is insufficient for the dose-response analysis in sacubitril-valsartan users due to the limited sample size. Lastly, this retrospective cohort study with a limited sample size may introduce potential selection biases, and further well-designed prospective studies are warranted to validate our findings.

## Conclusion

In conclusion, use of sacubitril-valsartan is associated with a significantly decreased risk of ineffictive ECV compared with non-users in patients with persistent AF. The findings of the present study indicate the potential value of sacubitril-valsartan in the rhythm-control management for persistent AF.

## Data Availability Statement

The raw data supporting the conclusions of this article will be made available by the authors, without undue reservation.

## Ethics Statement

According to International Ethical Guidelines for Health-Related Research Involving Humans, The Research Ethics Committee of the Second Affiliated Hospital of Chongqing Medical University reviewed and approved the study and granted a waiver of written informed consent.

## Author Contributions

QC, YC, FQ, HD, and YY: concept and design. QC, YC, FQ, CG, BZ, NW, MX, ZO, WZ, and BC: acquisition, analysis, or interpretation of data. QC and YC: drafting of the manuscript. HD, MX, ZL, and YY: critical revision of the manuscript. All authors contributed to the article and approved the submitted version.

## Conflict of Interest

The authors declare that the research was conducted in the absence of any commercial or financial relationships that could be construed as a potential conflict of interest.

## Publisher’s Note

All claims expressed in this article are solely those of the authors and do not necessarily represent those of their affiliated organizations, or those of the publisher, the editors and the reviewers. Any product that may be evaluated in this article, or claim that may be made by its manufacturer, is not guaranteed or endorsed by the publisher.

## References

[1] Zhang YY Qiu C Davis PJ Jhaveri M Prystowsky EN Kowey P Predictors of progression of recently diagnosed atrial fibrillation in registry on cardiac rhythm disorders assessing the control of atrial fibrillation (recordaf)-united states cohort. *Am J Cardiol.* (2013) 112:79–84. 10.1016/j.amjcard.2013.02.056 23561591

[2] Kirchhof P Camm AJ Goette A Brandes A Eckardt L Elvan A Early rhythm-control therapy in patients with atrial fibrillation. *N Engl J Med.* (2020) 383:1305–16. 10.1056/NEJMoa2019422 32865375

[3] Hindricks G Potpara T Dagres N Arbelo E Bax JJ Blomström-Lundqvist C 2020 ESC guidelines for the diagnosis and management of atrial fibrillation developed in collaboration with the european association for cardio-thoracic surgery (EACTS). *Eur Heart J.* (2021) 42:373–498. 10.1093/eurheartj/ehaa612 32860505

[4] Pisters R Nieuwlaat R Prins MH Le Heuzey JY Maggioni AP Camm AJ Clinical correlates of immediate success and outcome at 1-year follow-up of real-world cardioversion of atrial fibrillation: the euro heart survey. *Europace.* (2012) 14:666–74. 10.1093/europace/eur406 22223715

[5] Müssigbrodt A John S Kosiuk J Richter S Hindricks G Bollmann A. Vernakalant-facilitated electrical cardioversion: comparison of intravenous vernakalant and amiodarone for drug-enhanced electrical cardioversion of atrial fibrillation after failed electrical cardioversion. *Europace.* (2016) 18:51–6. 10.1093/europace/euv194 26056189 PMC7108474

[6] Um KJ McIntyre WF Healey JS Mendoza PA Koziarz A Amit G Pre- and post-treatment with amiodarone for elective electrical cardioversion of atrial fibrillation: a systematic review and meta-analysis. *Europace.* (2019) 21:856–63. 10.1093/europace/euy310 30875422

[7] Wijffels MC Kirchhof CJ Dorland R Allessie MA. Atrial fibrillation begets atrial fibrillation. A study in awake chronically instrumented goats. *Circulation.* (1995) 92:1954–68. 10.1161/01.cir.92.7.1954 7671380

[8] Allessie M Ausma J Schotten U. Electrical, contractile and structural remodeling during atrial fibrillation. *Cardiovasc Res.* (2002) 54:230–46. 10.1016/s0008-6363(02)00258-4 12062329

[9] Nattel S Li D. Ionic remodeling in the heart: pathophysiological significance and new therapeutic opportunities for atrial fibrillation. *Circ Res.* (2000) 87:440–7. 10.1161/01.res.87.6.440 10988234

[10] Efremidis M Alexanian IP Oikonomou D Manolatos D Letsas KP Pappas LK Predictors of atrial fibrillation recurrence in patients with long-lasting atrial fibrillation. *Can J Cardiol.* (2009) 25:e119–24. 10.1016/s0828-282x(09)70070-4 19340356 PMC2706771

[11] Raitt MH Volgman AS Zoble RG Charbonneau L Padder FA O’Hara GE Prediction of the recurrence of atrial fibrillation after cardioversion in the atrial fibrillation follow-up investigation of rhythm management (affirm) study. *Am Heart J.* (2006) 151:390–6. 10.1016/j.ahj.2005.03.019 16442905

[12] Kumagai K Nakashima H Urata H Gondo N Arakawa K Saku K. Effects of angiotensin ii type 1 receptor antagonist on electrical and structural remodeling in atrial fibrillation. *J Am Coll Cardiol.* (2003) 41:2197–204. 10.1016/s0735-1097(03)00464-9 12821247

[13] Shi Y Li D Tardif JC Nattel S. Enalapril effects on atrial remodeling and atrial fibrillation in experimental congestive heart failure. *Cardiovasc Res.* (2002) 54:456–61. 10.1016/s0008-6363(02)00243-2 12062350

[14] Sepehri Shamloo A Bollmann A Dagres N Hindricks G Arya A. Natriuretic peptides: biomarkers for atrial fibrillation management. *Clin Res Cardiol.* (2020) 109:957–66. 10.1007/s00392-020-01608-x 32002634

[15] Sezai A Iida M Yoshitake I Wakui S Osaka S Kimura H Carperitide and atrial fibrillation after coronary bypass grafting: the nihon university working group study of low-dose hanp infusion therapy during cardiac surgery trial for postoperative atrial fibrillation. *Circ Arrhythm Electrophysiol.* (2015) 8:546–53. 10.1161/circep.113.001211 25840580

[16] Ferrari R Cardoso J Fonseca MC Aguiar C Moreira JI Fucili A Arnis: balancing “the good and the bad” of neuroendocrine response to hf. *Clin Res Cardiol.* (2020) 109:599–610. 10.1007/s00392-019-01547-2 31531687

[17] Solomon SD Zile M Pieske B Voors A Shah A Kraigher-Krainer E The angiotensin receptor neprilysin inhibitor lcz696 in heart failure with preserved ejection fraction: a phase 2 double-blind randomised controlled trial. *Lancet.* (2012) 380:1387–95. 10.1016/s0140-6736(12)61227-6 22932717

[18] Li LY Lou Q Liu GZ Lv JC Yun FX Li TK Sacubitril/valsartan attenuates atrial electrical and structural remodelling in a rabbit model of atrial fibrillation. *Eur J Pharmacol.* (2020) 881:173120. 10.1016/j.ejphar.2020.173120 32325147

[19] Gubelli S Caivano M. Case of a patient with heart failure, dilated cardiomyopathy and atrial fibrillation treated with sacubitril/valsartan. *Curr Med Res Opin.* (2019) 35:19–22. 10.1080/03007995.2019.1598703 30895821

[20] De Vecchis R Paccone A Di Maio M. Upstream therapy for atrial fibrillation prevention: the role of sacubitril/valsartan. *Cardiol Res.* (2020) 11:213–8. 10.14740/cr1073 32595805 PMC7295563

[21] De Vecchis R Paccone A Di Maio M. Favorable effects of sacubitril/valsartan on the peak atrial longitudinal strain in patients with chronic heart failure and a history of one or more episodes of atrial fibrillation: a retrospective cohort study. *J Clin Med Res.* (2020) 12:100–7. 10.14740/jocmr4076 32095179 PMC7011939

[22] van Delden JJ van der Graaf R. Revised cioms international ethical guidelines for health-related research involving humans. *JAMA.* (2017) 317:135–6. 10.1001/jama.2016.18977 27923072

[23] Russo V Bottino R Rago A Papa AA Liccardo B Proietti R The effect of sacubitril/valsartan on device detected arrhythmias and electrical parameters among dilated cardiomyopathy patients with reduced ejection fraction and implantable cardioverter defibrillator. *J Clin Med.* (2020) 9:1111. 10.3390/jcm9041111 32294983 PMC7230317

[24] de Diego C González-Torres L Núñez JM Centurión Inda R Martin-Langerwerf DA Sangio AD Effects of angiotensin-neprilysin inhibition compared to angiotensin inhibition on ventricular arrhythmias in reduced ejection fraction patients under continuous remote monitoring of implantable defibrillator devices. *Heart Rhythm.* (2018) 15:395–402. 10.1016/j.hrthm.2017.11.012 29146274

[25] Suo Y Yuan M Li H Zhang Y Li Y Fu H Sacubitril/valsartan improves left atrial and left atrial appendage function in patients with atrial fibrillation and in pressure overload-induced mice. *Front Pharmacol.* (2019) 10:1285. 10.3389/fphar.2019.01285 31736759 PMC6830387

[26] Januzzi JL Jr. Prescott MF Butler J Felker GM Maisel AS McCague K Association of change in n-terminal pro-b-type natriuretic peptide following initiation of sacubitril-valsartan treatment with cardiac structure and function in patients with heart failure with reduced ejection fraction. *JAMA.* (2019) 322:1085–95. 10.1001/jama.2019.12821 31475295 PMC6724151

[27] Desai AS Solomon SD Shah AM Claggett BL Fang JC Izzo J Effect of sacubitril-valsartan vs enalapril on aortic stiffness in patients with heart failure and reduced ejection fraction: a randomized clinical trial. *JAMA.* (2019) 322:1077–84. 10.1001/jama.2019.12843 31475296 PMC6749534

[28] Okutucu S Fatihoglu SG Sabanoglu C Bursa N Sayin BY Aksoy H Effects of angiotensin receptor neprilysin inhibition on p-wave dispersion in heart failure with reduced ejection fraction. *Herz.* (2019) 46:69–74. 10.1007/s00059-019-04872-4 31796977

[29] Fujimoto Y Yodogawa K Maru YJ Oka E Hayashi H Yamamoto T Advanced interatrial block is an electrocardiographic marker for recurrence of atrial fibrillation after electrical cardioversion. *Int J Cardiol.* (2018) 272:113–7. 10.1016/j.ijcard.2018.07.135 30072150

[30] Fujimoto Y Yodogawa K Takahashi K Tsuboi I Hayashi H Uetake S Noninvasive evaluation of reverse atrial remodeling after catheter ablation of atrial fibrillation by p wave dispersion. *Heart Vessels.* (2017) 32:1375–81. 10.1007/s00380-017-1008-1 28631077

[31] Madrid AH Bueno MG Rebollo JM Marín I Peña G Bernal E Use of irbesartan to maintain sinus rhythm in patients with long-lasting persistent atrial fibrillation: a prospective and randomized study. *Circulation.* (2002) 106:331–6. 10.1161/01.cir.0000022665.18619.83 12119249

[32] Ueng KC Tsai TP Yu WC Tsai CF Lin MC Chan KC Use of enalapril to facilitate sinus rhythm maintenance after external cardioversion of long-standing persistent atrial fibrillation. Results of a prospective and controlled study. *Eur Heart J.* (2003) 24:2090–8. 10.1016/j.ehj.2003.08.014 14643269

[33] Hubers SA Brown NJ. Combined angiotensin receptor antagonism and neprilysin inhibition. *Circulation.* (2016) 133:1115–24. 10.1161/circulationaha.115.018622 26976916 PMC4800749

[34] Mullens W Martens P. Exploiting the natriuretic peptide pathway to preserve glomerular filtration in heart failure. *JACC Heart Fail.* (2018) 6:499–502. 10.1016/j.jchf.2018.02.017 29655826

[35] Goetze JP Bruneau BG Ramos HR Ogawa T de Bold MK de Bold AJ. Cardiac natriuretic peptides. *Nat Rev Cardiol.* (2020) 17:698–717. 10.1038/s41569-020-0381-0 32444692

[36] Carluccio E Dini FL Bitto R Ciccarelli M Correale M D’Agostino A Benefit from sacubitril/valsartan is associated with hemodynamic improvement in heart failure with reduced ejection fraction: an echocardiographic study. *Int J Cardiol.* (2022) 350:62–8. 10.1016/j.ijcard.2022.01.004 34998946

[37] Dini FL Carluccio E Bitto R Ciccarelli M Correale M D’Agostino A Echocardiographically defined haemodynamic categorization predicts prognosis in ambulatory heart failure patients treated with sacubitril/valsartan. *ESC Heart Fail.* (2022) 9:1107–17. 10.1002/ehf2.13779 35122477 PMC8934975

[38] Marchese P Bursi F Delle Donne G Malavasi V Casali E Barbieri A Indexed left atrial volume predicts the recurrence of non-valvular atrial fibrillation after successful cardioversion. *Eur J Echocardiogr.* (2011) 12:214–21. 10.1093/ejechocard/jeq176 21149290

[39] Burstein B Nattel S. Atrial fibrosis: mechanisms and clinical relevance in atrial fibrillation. *J Am Coll Cardiol.* (2008) 51:802–9. 10.1016/j.jacc.2007.09.064 18294563

[40] Crijns HJ Weijs B Fairley AM Lewalter T Maggioni AP Martín A Contemporary real life cardioversion of atrial fibrillation: results from the multinational rhythm-af study. *Int J Cardiol.* (2014) 172:588–94. 10.1016/j.ijcard.2014.01.099 24556445

[41] Toso E Iannaccone M Caponi D Rotondi F Santoro A Gallo C Does antiarrhythmic drugs premedication improve electrical cardioversion success in persistent atrial fibrillation? *J Electrocardiol.* (2017) 50:294–300. 10.1016/j.jelectrocard.2016.12.004 28069273

